# Dental Anomalies in Primary Dentition among Arabian Children: A Hospital-Based Study

**DOI:** 10.3390/children11030366

**Published:** 2024-03-19

**Authors:** Sreekanth Kumar Mallineni, Abdullah Alassaf, Basim Almulhim, Sara Alghamdi

**Affiliations:** 1 Pediatric Dentistry, Dr. Sulaiman Al Habib Hospital, Ar Rayyan, Riyadh 14212, Saudi Arabia; 2 Division for Globalization Initiative, Liaison Center for Innovative Dentistry, Graduate School of Dentistry, Tohoku University, Sendai 980-8575, Japan; 3 Department of Preventive Dental Sciences, College of Dentistry, Majmaah University, Al Majmaah 11952, Saudi Arabia

**Keywords:** dental anomaly, children, primary dentition, supernumerary teeth, taurodontism

## Abstract

An observational study was carried out in a teaching hospital in Saudi Arabia to determine the occurrence of dental anomalies among Arabian children. The study included children of Saudi nationality with primary teeth. The study assessed the prevalence of dental anomalies in their primary dentition. The assessment and data collection were conducted by a single examiner, utilizing clinical examination and intra-oral radiographs. A comparative analysis was conducted to examine dental anomalies in relation to gender (boys and girls) and arch type (maxillary and mandibular). In addition, the study explored the occurrence of gender-specific dental anomalies depending on arch type. The data analysis was conducted using IBM Statistics (version 21.0) with a significance level of *p* < 0.05. In total, there were 245 children included in the final analysis. The study population consisted of boys (66%) and girls (34%), with an average age of 4.87 ± 0.9 years. Taurodontism was the most prevalent dental abnormality, occurring in 2.8% of the individuals in the study sample. The study sample exhibited hypodontia in 2%, supernumerary teeth in 2.4%, double teeth in 2%, and microdontia in 1.2%. Talon cusp and macrodontia have a relatively low incidence of 0.4%. Boys exhibit supernumerary teeth, microdontia, macrodontia, talon cusp, and taurodontism, whereas hypodontia and double teeth were more frequent in girls.

## 1. Introduction

Morphological and numerical abnormalities are the most prevalent forms of developing dental malformations. These anomalies develop due to disturbances that occur during various stages of tooth development [1]. The relevant data regarding the frequency and dispersion of certain dental abnormalities may not be voluntarily revealed during regular clinical examinations unless specifically mentioned by the patient as their main concern [2,3,4]. To assist in the diagnosis of associated asymptomatic teeth with dental anomalies, it is necessary to perform a comprehensive clinical and radiographic evaluation of the complete oral cavity [5,6]. The most frequent complications of dental anomalies include ectopic position, malocclusion, impactions, and difficulty in dental procedures like restoration, surgery, etc. [4,6,7]. Various reasons during different stages of tooth development can cause anomalies in tooth size, shape, structure, and position, eventually becoming evident as dental anomalies. Tooth number anomalies are defined by the occurrence of either an excess or an absence of teeth compared to the normal complement of primary or permanent dentitions. These abnormalities arise from abnormalities in the dental lamina or tooth germ during the process of tooth development. The majority of the studies reported in the literature focused on supernumerary teeth, hypodontia, double teeth, talon cusp, macrodontia, microdontia, dens invaginatus, and taurodontism. These anomalies can yield numerous disturbances in both the dentitions and also in the dental arch lengths and occlusions [8,9,10]. Timely identification and appropriate treatment planning at an initial phase are required to avoid possible complications that can occur with dental abnormalities [8,9,10].

Researchers have not thoroughly established the specific etiopathogenesis in the literature and both environmental and genetic factors play a major role in the development of dental anomalies in developing teeth [8,9,10]. Both developmental and acquired dental disorders affect the primary and permanent dentitions. The presence of dental anomalies in the dental arch may cause significant problems, including the structure and function of teeth. As a result, a thorough examination of the factors that control their growth needs to be established. Research indicates that certain hereditary variables can lead to tooth anomalies during dentition development in both arches. Furthermore, disturbances during tooth development can cause variations in the shape, size, and number of teeth [7,9], and the position of the tooth bud in the developing stage influences both the permanent and deciduous dentitions of both jaws [10]. Many epidemiological studies have examined primary dentition features in different populations. There are various studies [11,12,13,14,15,16,17,18,19,20,21,22,23,24,25] published in the literature on dental anomalies from Saudi Arabia. The majority of researchers are focused on mixed dentition and permanent dentition, and some of the researchers reported dental anomalies in the orthodontic population and individuals with clefts. It is imperative to distinguish and subsequently provide appropriate treatment for these dental anomalies to avoid potential complications. Dental abnormalities in Arab children with primary dentition have not been studied. Nonetheless, there is a need to study dental anomalies in primary dentition. Therefore, the purpose of the study was to assess the prevalence of dental anomalies in primary dentition among Arabian schoolchildren attending a teaching hospital.

## 2. Materials and Methods

The Deanship of Scientific Research at Majmaah University, Almajmaah, Kingdom of Saudi Arabia, authorized the study proposal. The study was carried out between October 2020 and April 2022. The investigation was conducted at Majmaah University in Al-Zulfi, Saudi Arabia, specifically at the Department of Pediatric Dentistry, which is part of the College of Dentistry. The study comprised only children who sought treatment for dental problems at the university hospital. Obtaining informed consent from all parents and guardians of the children who participated in the study was ensured. A comprehensive clinical and radiographic assessment of the complete oral cavity was conducted to detect dental anomalies. Healthy children of Saudi origin with primary teeth were included in the study. Children who did not meet the following criteria were excluded from the study: non-Saudi children, intellectual disabilities, cleft lip and/or palates, systemic diseases, related syndromes, and parental refusal to provide consent. A single investigator examined the oral cavity of each child to evaluate the dental anomalies. The dental anomalies were categorized based on appearance and characteristics [26,27,28], which included the number (hypodontia [29] and supernumerary teeth [30]), size and shape (double teeth [31], microdontia [27,29,32], macrodontia [27,29,32], and taurodontism [33]), and structure (talon cusps [34], dens invaginatus [35]). To estimate the reproducibility of the diagnosis, 25 radiographs were selected randomly and examined once again separately by the same operator. Kappa statistics showed excellent intra-examiner reliability (K = 0.93). The intra-oral bitewing radiographs and additional intra-oral radiographs based on necessity were considered for radiographic evaluation. The clinical and radiographic records of the children who participated in the study were used to investigate the prevalence of each dental anomaly.

The diagnostic criteria used to identify the dental anomalies in the study were as mentioned below:

Hypodontia is the congenital absence of one or more teeth clinically and radiographically [29].

Supernumerary tooth is any tooth or odontogenic structure that is formed from a tooth germ more than the normal number for any given region of the dental arch [30].

Double teeth involve joining two developing tooth germs that result in a larger-sized tooth with two distinct tooth germs [31].

Talon cusp is an extra cusp-like structure that extends from the cementoenamel junction towards the incisal edge in the anterior teeth [34].

Dens invaginatus is an invagination of enamel, and dentine towards the pulp [35].

Microdontia is a rare dental anomaly in which teeth appear smaller than the normal complement [27,29,32].

Macrodontia is a rare dental anomaly where teeth appear larger than the normal complement [27,29,32].

Taurodontism is a developmental anomaly characterized by a large pulp chamber in a multirooted tooth, along with an apical displacement of the pulp floor and bifurcation of the root [33].

Statistical Analysis:

A comparison of the occurrence of dental anomalies based on gender and arch was performed; additionally, the gender- and arch-based occurrence of dental anomalies was compared based on arch type. To determine whether there were any differences between the groups, categorical data were subjected to the chi-square test. To evaluate the significant association among the dental anomalies, the Spearman rank correlation coefficient was used. All the descriptive statistics were carried out using IBM SPSS Statistics for Windows (Version 21.0. Armonk, NY, USA: IBM Corp.), and, at a probability value of 0.05, the significance of every statistical test was predetermined.

## 3. Results

Overall, 245 children were available for the analysis based on the inclusion and exclusion criteria. Among the study population, the majority of them were boys (66%) and girls (34%) with the mean age of the study population being 4.87 ± 0.9 years. Table 1 shows that there were only seven dental abnormalities that were observed in Arabian children. The prevalence rates of dental anomalies including hypodontia, supernumerary teeth, double teeth, talon cusp, microdontia, macrodontia, and taurodontism were as follows: 2.4%, 2%, 2%, 0.4%, 1.2%, 0.4%, and 2.8%. Taurodontism is one of the most common dental anomalies, with a prevalence of 2.8%. However, only 0.4% of children were observed with talon cusp and macrodontia. No case of dens invaginatus was found in the Arabian children who participated in the study.

The summary of the gender-based comparison of dental anomalies in the study population was summarized in Table 2. The comparison of the occurrence of dental anomalies based on gender revealed that the prevalence of supernumerary teeth and microdontia is higher in boys than in girls. On the other hand, the prevalence of hypodontia, double teeth, and taurodontism is higher in girls than in boys who have these conditions. The only individuals who exhibited talon cusp and macrodontia were boys. According to the statistical analysis, it was determined that none of the gender-based comparisons concerning dental malformations were statistically significant (*p* < 0.05).

Hypodontia, supernumerary teeth, double teeth, and talon cusps were taken into consideration for the arch-based occurrence of dental anomalies. The mandibular arch accounted for 52 percent of all total anomalies, while the maxillary arch accounted for 48 percent of all dental anomalies observed (Table 3). The present study found that the maxillary arch has a higher prevalence of supernumerary teeth and talon cusps, while the mandibular arch more commonly exhibits hypodontia and double teeth. There was no statistically significant difference between any of the comparisons (*p* < 0.05).

Table 4 summarizes the gender-based comparison of the arch-based distribution of dental anomalies in primary dentition. None of the comparisons of the occurrence of gender-based dental anomalies in both arches showed statistical significance (*p* > 0.05). The majority of the supernumerary teeth were observed in boys and were located in the maxillary arch. Both boys and girls exhibited the distribution of hypodontia in both the maxillary and mandibular arches. Double teeth were observed to be located in the maxillary arch in boys and the mandibular arch in girls. The study population did not observe supernumerary teeth, or talon cusps, in the mandibular arch.

The most prevalent type of supernumerary teeth was supplemental tooth type, which accounted for 5% of all cases. Other common types of missing teeth included hypodontia in the maxillary arch, which resulted in bilateral missing lateral incisors, and mandibular arch lateral incisors. Both the lateral incisor and the canine are examples of double teeth that are quite prevalent in the mandibular arch. Overall, 20 children (8.1%) were observed with dental anomalies in the study population. Among them, 55% of dental anomalies in boys and 45% in girls (Table 5) had non-statistically significant difference (*p* > 0.05). A total of 35% of boys were observed with at least one dental anomaly while 10% of girls were found with one dental anomaly. A total of 30% of girls observed at least 2 dental anomalies while 15% of the girls observed two dental anomalies. Five percent of the boys and girls were observed with three or more dental anomalies.

Supplemental incisors were the common type of supernumerary in primary dentition. The mandibular lateral incisor was the commonly missing tooth in primary dentition. On the other hand, the central incisor and the lateral incisor were examples of double teeth in the maxillary arch and only a case of talon cusp was observed in the maxillary central incisor. The Spearman rank correlation coefficient found a positive correlation between taurodontism, hypodontia, double teeth, and microdontia (Table 6). None of the studied dental anomalies were found to be associated with supernumerary teeth, macrodontia, or talon cusp. Hypodontia showed a positive correlation with taurodontism and microdontia, with a statistically significant difference (*p* < 0.001). Similarly, taurodontism should have a positive correlation with the double tooth, hypodontia, and microdontia, with a statistically significant difference (*p* < 0.001).

## 4. Discussion

During routine dental examinations of children, pediatric dentists often detect dental anomalies in primary dentition, which can lead to orthodontic problems such as aesthetic issues, spacing or crowding of teeth, deviation of the midline, increased caries risk, and loss of arch length in children [4,5,6]. The present observational study evaluated the prevalence of dental anomalies and the gender-based and arch-based occurrence of dental anomalies in Arabic children attending the faculty of dentistry in Saudi Arabia. Various researchers conducted prevalence-based studies in Saudi Arabia that involved the participation of children in both mixed dentistry and permanent dentistry for their analysis [11,12,13,14,15,16,17,18,19,20,21,22,23,24,25]. Nonetheless, the theme included children with primary dental care from Saudi Arabia. Overall, the prevalence of dental anomalies in the present study population was 8.1%. The observed prevalence figures were almost equal to studies reported from the United States of America [36] and Japan [37]. The prevalence figures were higher than the studies reported from Sweden [38], Brazil [39], the United Kingdom [40], Iceland [41], New Zealand [42], and Belgium [43]. The numbers were relatively high when compared to other published studies, which may be because this study sample has been recruited from a hospital. Reports indicate that children with problems visit dental hospitals, and the sample size is also quite low. The higher figures of occurrence of dental anomalies might warrant a further study among the Arabian population with a large sample of children. Studies have reported that there is a correlation between dental anomalies in the primary and permanent dentition [9,12,24]. In more than 50% of these cases, dental anomalies in primary dentition have a marked effect on their successors and the developing occlusion [24,25,26].

The prevalence of supernumerary teeth in the present study sample was approximately 2%. The prevalence figures of the present study were slightly higher compared to the published studies [7,36,37,38,39,40,41,42,43], which were less than 1%. On the other hand, they were slightly lower than in the Hong Kong study [44], and the prevalence was similar to that reported in an American study [45] in primary and mixed children: 2%. In the majority of the studies, this numerical anomaly was more common in boys than girls; similarly, the present study reported male predilection of supernumerary teeth (*p* < 0.05). In the present study, a supplemental incisor was a commonly observed supernumerary-type tooth in the anterior maxillary region. The findings were in agreement with the literature [26,44] on the occurrence of supernumerary teeth in the anterior region of the maxillary arch. This dental anomaly is more commonly reported in the maxillary arch than in the mandibular arch [45,46]. Correspondingly, all the supernumerary teeth in the present study were observed in all the cases in the maxillary arch. The study findings were in agreement with the published literature [47,48], which showed that boys had more supernumerary teeth than girls, with no statistically significant difference (*p* < 0.05).

Based on the prior reported studies, the prevalence of hypodontia ranged from 2.2% to 9.7% among children in Saudi Arabia [11,12,13,14,15,16,17,18,19,20,21,22,23,24,25]. In the present study, the authors observed hypodontia at 2.4% of the prevalence in primary dentition. Similarly, a Japanese study [37] reported almost the same prevalence figures for hypodontia. Some of the earlier studies [7,37,44,45,49] reported a hypodontia prevalence of less than 2%. Conversely, few of the earlier studies [44,45] reported more than the present study findings. The Hong Kong study [44] and the American study [45] reported a 4.1% and 4.6% prevalence of hypodontia in Southern Chinese and American children, respectively. Subsequently, Chen et al.’s [50] study reported hypodontia with a 2% prevalence among Taiwanese children. The present study showed girls had a higher predilection for hypodontia than boys, with non-statistically significant results (*p* < 0.05). This is in agreement with the majority of the studies published in the literature. The mandibular lateral incisor was more commonly missing in the study population. Hypodontia was more common in the mandibular arch than the maxillary arch, with no significant difference (*p* < 0.05). However, the majority of the published literature reported that hypodontia is more common in the maxillary arch than the mandibular arch [45,51,52].

The prevalence of the double teeth in the present study was 2%, and these prevalence figures were accordingly higher than in studies reported from India [7], Sweden [37], Brazil [39], the United Kingdom [40], Iceland [41], New Zealand [42], and Belgium [43]. Nonetheless, the studies from Hong Kong [44], Taiwan [50], and Japan [37] observed 4.1%, 3%, and 4.1%, respectively. This dental anomaly was reported to be more prevalent in Chinese and Japanese [37] ethnic groups than in Caucasians [45]. In the present study, both gemination and fusion were considered double teeth, and additional radiographs were not taken to establish the difference among the double teeth [45]. The published literature reports that this dental anomaly occurs predominantly in the anterior region of the mandibular arch and often involves canines and lateral incisors [53,54,55]. Studies from Hong Kong [44], Taiwan [50], and Japan [37] observed that the double tooth involves central and lateral incisors in the maxillary arch and canines and a lateral incisor in the mandibular incisor. Similarly, in the present study, all the double teeth were observed in the anterior region, and the double tooth involved central and lateral incisors in both maxillary and mandibular arches. Even though this is a coincidental and novel finding in the study, further confirmation is required with a large sample. In the present study, double teeth were more common in the mandibular arch with no statistically significant difference. This is also in agreement with the findings observed in the literature by a few authors [53,55,56]. The girls were observed to have more double teeth than the boys, with no statistically significant difference.

The prevalence of macrodontia was 2.4% in the study population. Microdontia can be true generalized, relatively generalized, or localized macrodontia [26]. The present study found 1.2% truly generalized microdontia and 1.2% localized microdontia with peg laterals. The majority of reported studies on microdontia reported less than 1% [7,36,37,39,45,46]. Conversely, a study from Hong Kong reported 6.3% among the southern Chinese children [44]. These authors observed microdontia more frequently in the maxillary arch compared to the mandibular arch. Similarly, in the present study, microdontia was more commonly observed in the maxillary arch, with no statistically significant difference. Previous studies have reported a higher prevalence of microdontia in the primary maxillary canines among Hong Kong schoolchildren [44]. In the present study, the primary lateral incisor was more significant than the microdontia. In the present study, microdontia was more frequent in boys compared to girls, with statistically no significant difference. However, these findings are not in agreement with the findings reported in the literature from the British study [57].

The various types involved in macrodontia were generalized (true or relatively) or localized [26]. In the present study, only one case was reported with macrodontia. Localized macrodontia was observed in the maxillary arch with central incisors as the only case in the present study with a 0.4% prevalence. Otherwise, macrodontia in primary dentition is not frequently described in the published literature [57,58,59]. The prevalence of macrodontia in primary teeth has been studied in three studies reported from the United Kingdom [40], Japan [59], and Hong Kong [44]. The reported prevalence of macrodontia in Japanese children was 3.5%, and in southern Chinese children, it was 2.3%. In the present case, the only reported case was in a male patient involving maxillary central incisors. The gender-based and arch-based comparisons were not statistically significant (*p* < 0.05) in the present study. The findings were in agreement with a consequent British study [57,58] that found macrodontia was more common in boys than girls. In the present study, the authors did not measure the mesiodistal width of the subject; the macrodontia was considered based on observations. To be considered for the Japanese study, a tooth had to be at least three and a half standard deviations larger than the average size of the mesiodistal teeth for the genders involved. In the present study, macrodontia was diagnosed based on Chowdary’s criteria [32].

There are extremely few published studies on the talon cusp in people without clefts or syndromes because of the extraordinary rarity of this defect in the primary dentition. The majority of studies reported in the literature on the prevalence of talon cusps have been involved more frequently in permanent dentition than primary dentition. In the present study, only one case of talon cusp was observed with a prevalence of 0.4%. Prior studies reported a comparable prevalence of talon cusps in primary dentistry. Among those, a Japanese study reported 0.6% [59], whereas a study involving South Chinese children reported a 0.5% prevalence of talon cusps in primary dentistry. However, an Indian study [7] reported a 0.04% prevalence. According to al-Omari and colleagues [60], the talon cusp is more frequent in boys than girls in the primary dentition by a ratio of 3.5:1. Another literature review on talon cusps in primary dentition by Lee and his co-workers [61] stated that boys are more frequently affected than girls in primary dentition, with a ratio of 1.8:1. The higher tendency in boys may indicate that the genes that cause talon cusps in primary teeth are different in boys and girls [62,63]. In the present study, the only case reported was in a male subject involving a primary central incisor.

The authors of the present study observed taurodontism in 2.8% of the study population. A Japanese study reported a 0.5% prevalence of dental anomalies in primary molars, compared to 0.08% in northern Europeans [64,65]. According to Jørgensen [66], the population of modern Danes has a prevalence of 9.0%, which is an extraordinarily high percentage. The study conducted with Swedish children [57] found conclusions that contradict the current findings. The present study observed female predilection for this dental anomaly with no statistical significance. The findings were in agreement with prior studies reported in the literature [65,66]. The taurodontism can be hypo, mesio, or hyper. However, in the present study, this anomaly was not evaluated based on type.

Hypodontia showed a positive correlation with taurodontism and microdontia, with a statistically significant difference (*p* < 0.001). Similarly, taurodontism should have a positive correlation with the double tooth, hypodontia, and microdontia, with a statistically significant difference (*p* < 0.001). A recent systematic review [67] reported that impacted teeth and taurodontism are the most frequently associated dental anomalies with hypodontia in permanent teeth. In the present study, a correlation was found among the primary dentitions. Even though the findings are similar, the present study focused on dental anomalies in primary dentition, while the review explored dental anomalies in permanent dentistry. An Italian study [68] found no association between hypodontia and taurodontism, and the authors studied dental anomalies among permanent dentitions. Brook et al. [57] postulated that hypodontia and microdontia are correlated, which was evident in the study. In the study, the authors reported that supernumerary and microdontia are common in boys, and hypodontia and microdontia are frequent in girls. The present study findings were in agreement with Brook et al. Two Australian studies [69,70] found an association between taurodontism and hypodontia in permanent dentistry; however, the study concentrated on children with primary dentistry. Hence, the findings were not comparable. None of the studied dental anomalies were found to be associated with supernumerary teeth, macrodontia, or talon cusps.

Researchers from various countries reported the prevalence of dental anomalies in both primary and permanent dentitions, with some also including children with mixed dentition [70,71,72,73,74,75,76]. To build a realistic interaction between investigators and assure accurate and consistent data recording, it is necessary to determine the many features and diagnostic components of these dental anomalies and qualities. There have been fifteen prior studies [11,12,13,14,15,16,17,18,19,20,21,22,23,24,25] reported from the Kingdom of Saudi Arabia; most of the studies focused on mixed dentition and permanent dentition. One of these studies described the correlation of dental anomalies between primary and permanent dentitions [25]. Nevertheless, documented research indicates that the Arabian exhibits variation and needs to analyze the dental anomalies in primary dentition. Ultimately, this will enhance a child’s clinical management of a specific dental anomaly. Early detection of the conditions that predispose one to develop a malocclusion in permanent dentition is very important [74,75,76]. The study limitations include a very small sample size, all the subjects being taken from a teaching hospital, and the only radiographs used for analysis. However, the prior authors reported dental casts, panoramic radiographs, intra-oral radiographs, and clinical examinations. Practically, taking panoramic radiographs of all the children was not possible, and this is unjustifiable. Hence, in the present study, the authors used clinical examination, radiographic examination of the bitewings, and additional intra-oral radiographs to confirm the presence of dental anomalies. The study did not evaluate the correlation among the various anomalies; this is considered a limitation. The present study did not assess the distribution and correlation of dental anomalies with their successors; this can also be considered a limitation of the study. One of the recent studies evaluated dental anomalies in children with primary dentition from Saudi Arabia. Although this study collected data to evaluate the prevalence of dental anomalies in Arabian children with primary dentition, researchers need to conduct more studies to improve data on the prevalence of primary dentition features and enhance our understanding of the anthropological significance of identified dental anomalies.

## 5. Conclusions

Within the limitations, the prevalence of dental anomalies among Arabian children, including hypodontia, supernumerary teeth, double tooth, talon cusp, microdontia, macrodontia, and taurodontism, was observed in 2.4%, 2%, 2%, 0.4%, 1.2%, 0.4%, and 2.8%, respectively, of Arabian children. Supernumerary teeth, microdontia, macrodontia, talon cusp, and taurodontism were observed with male predilection whereas hypodontia and double tooth showed female predilection in the present study.

## Figures and Tables

**Table 1:** The overall prevalence of dental anomalies in primary dentition.

Dental Anomalies	No of Children (N)	Prevalence (%)
Hypodontia	6	2.4
Supernumerary teeth	5	2
Double teeth	5	2
Talon cusp	1	0.4
Microdontia	3	1.2
Macrodontia	1	0.4
Taurodontism	7	2.8

**Table 2:** Gender-based comparison of dental anomalies.

Dental Anomalies	Boys N (%)	Girls N (%)	*p*-Value
Hypodontia	2(33.3%)	4 (66.7%)	0.91
Supernumerary teeth	4 (80%)	1 (20%)	0.497
Double teeth	2 (40%)	3 (60%)	0.221
Talon cusp	1 (100%)	0 (0)	0.16
Microdontia	2 (66.6%)	1 (33.3%)	0.972
Macrodontia	1 (100%)	0 (0)	0.47
Taurodontism	3(42.9%)	4 (57.1%)	0.15

**Table 3:** Arch-based occurrence of dental anomalies.

Dental Anomalies	Maxillary Arch (%)	Mandibular Arch (%)
Hypodontia	2 (33%)	4 (67%)
Supernumerary teeth	5 (100%)	0 (0)
Double teeth	2 (40%)	3 (60%)
Talon cusp	1 (100%)	0 (0)

**Table 4:** Gender-based comparison of the arch-based distribution of dental anomalies.

Dental Anomalies		Boys	Girls	*p*-Value
Hypodontia	Maxillary arch	1	1	0.178
	Mandibular arch	1	3	
Supernumerary teeth	Maxillary arch	4	1	0.497
	Mandibular arch	0	0	
Double teeth	Maxillary arch	2	0	0.33
	Mandibular arch	0	3	
Talon cusp	Maxillary arch	1	0	0.148
	Mandibular arch	0	0	

**Table 5:** Gender-based comparison of the distribution of dental anomalies in numbers.

Gender	1 (%)	2 (%)	3 or More (%)	Total (%)	*p*-Value
Boys	7 (35)	3 (15)	1 (5)	11(55)	
Girls	2 (10)	6 (30)	1 (5)	9(45)	>0.05
Total	9(45)	9 (45)	2 (10)	20 (100)	

**Table 6:** Associations between various dental anomalies in the study population.

		Hypodontia	Supernumerary Teeth	Double Teeth	Talon Cusp	Microdontia	Macrodontia	Taurosdontism
Hypodontia	Spearman’s rho	-						
	*p*-Value	-						
Supernumerary teeth	Spearman’s rho	−0.023	-					
	*p*-Value	0.722	-					
Double teeth	Spearman’s rho	−0.023	−0.021	-				
	*p*-Value	0.722	0.746	-				
Talon cusp	Spearman’s rho	−0.010	−0.009	0.009	-			
	*p*-Value	0.874	0.886	0.886	-			
Microdontia	Spearman’s rho	0.463 ***	0.016	−0.016	−0.007	-		
	*p*-Value	<0.001	0.802	0.802	0.912	-		
Macrodontia	Spearman’s rho	−0.010	−0.009	−0.009	−0.004	−0.009	-	
	*p*-Value	0.874	0.886	0.886	0.949	0.886	-	
Taurodontism	Spearman’s rho	0.448 ***	−0.025	0.495 ***	−0.011	0.426 ***	−0.011	-
	*p*-Value	<0.001	0.700	<0.001	0.864	<0.001	0.864	-

Spearman rank correlation coefficient. *p*-Value *p* < 0.05 (predetermined significance), *p* < 0.01 (predetermined significance), *** *p* < 0.001 (Highly significant) was considered as significant; - = not applicable.

## Data Availability

Data will be available upon request to the corresponding authors. The data are not publicly available in accordance with the consent provided by participants for the use of confidential data.
